# Magnetic switching of nanoscale antidot lattices

**DOI:** 10.3762/bjnano.7.65

**Published:** 2016-05-24

**Authors:** Ulf Wiedwald, Joachim Gräfe, Kristof M Lebecki, Maxim Skripnik, Felix Haering, Gisela Schütz, Paul Ziemann, Eberhard Goering, Ulrich Nowak

**Affiliations:** 1 Institute of Solid State Physics, Ulm University, Albert-Einstein-Allee 11, 89069 Ulm, Germany; 2 Faculty of Physics and Center for Nanointegration Duisburg-Essen (CENIDE), University of Duisburg-Essen, Lotharstr. 1, 47057 Duisburg, Germany; 3 Max-Planck-Institute for Intelligent Systems, Heisenbergstr. 3, 70569 Stuttgart, Germany; 4 Department of Physics, University of Konstanz, 78457 Konstanz, Germany; 5 IT4Innovations Centre, VSB Technical University of Ostrava, Czech Republic

**Keywords:** antidot lattice, first-order reversal curves, Kerr microscopy, magnetic nanostructures, magnetic switching, micromagnetic simulations, plasma etching, spin ice, X-ray microscopy

## Abstract

We investigate the rich magnetic switching properties of nanoscale antidot lattices in the 200 nm regime. In-plane magnetized Fe, Co, and Permalloy (Py) as well as out-of-plane magnetized GdFe antidot films are prepared by a modified nanosphere lithography allowing for non-close packed voids in a magnetic film. We present a magnetometry protocol based on magneto-optical Kerr microscopy elucidating the switching modes using first-order reversal curves. The combination of various magnetometry and magnetic microscopy techniques as well as micromagnetic simulations delivers a thorough understanding of the switching modes. While part of the investigations has been published before, we summarize these results and add significant new insights in the magnetism of exchange-coupled antidot lattices.

## Introduction

In nanotechnology, a widely used approach for tailoring physical properties on the nanometre length scale is the introduction of practically circular holes – so-called antidots – into thin films. Such antidots act as an inner surface of the materials leading to strong variations of optical [1–2], electrical [3–4], superconducting [5–6], or magnetic properties [7]. Nowadays, top-down approaches like e-beam lithography [8–9] or focused ion beam milling (FIB) [10] and bottom-up techniques based on the self-assembly of nanoscale spheres [2,11–12] allow precise control over diameter and distance of the antidots. In the present work, we make use of bottom-up nanosphere lithography in combination with reactive ion etching resulting in hexagonally arranged, non-close packed spherical masks for the production of magnetic antidot lattices.

In magnetic antidot films, the nanoscale periodic structure of holes introduces an in-plane shape anisotropy to the otherwise isotropic in-plane properties of polycrystalline or amorphous thin films. Additionally, the holes may act as artificial pinning sites for domain walls, blocking or even guiding their movement in specific directions during magnetic reversal [13–14]. Moreover, magnetic antidot lattices eventually lead to the formation of magnetic vortex structures [15].

In the extreme, the choice of large antidot diameters *d* > 0.75*a*, with the antidot distance *a,* has proven forming an artificial spin-ice system by geometric frustration [16]. Recently, local switching events in such artificial spin-ice systems have attracted much attention for the production of pairs of magnetic monopoles still obeying Maxwell equations [17]. At the other end of the tuneable antidot diameters producible by nanosphere lithography, small antidot dimensions with *d* < 0.1*a* were suggested for applications in magnonics, where spin waves are used to transmit and process information [18]. Using the periodicity of the antidot lattice, the spin wave dispersion is shaped by the introduction of gap states and such a magnonic crystal can correspondingly act as a band pass filter for spin waves [19].

The present contribution covers practical aspects on the production and characterisation of antidots while the second part elucidates the various switching modes for in-plane magnetised Fe, Co and Permalloy as well as for out-of-plane magnetised GdFe antidot films with different periodicities. We collect a series of previous results and complement them with new investigations for a thorough understanding of the switching modes in antidot lattices in this comprehensive contribution. We start the discussion with a technical section on the achievements and limitations of magnetic antidot arrays by bottom-up nanosphere lithography and specially developed characterisation and simulation tools. We show that the development of a proper spatially resolving magnetometry at a lateral resolution better than the structural grain size of the antidots based on magneto-optical Kerr (MOKE) microscopy is possible and the determination of interaction- and coercive field-distributions by fast MOKE related first-order reversal curves (FORC) is feasible. The hands-on application of micromagnetic simulations leads to a detailed understanding of the switching modes of specific sample geometries. We highlight that even anisotropic magnetoresistance curves can be simulated in good agreement with experiments.

In the second part, we first focus on the detailed discussion of the magnetic properties of in-plane magnetized antidot films. Integral magnetometry averaging over all in-plane angles of the antidots with respect to the external field proves that the system is highly dominated by the local shape anisotropy introduced by the hole sites. We identify two distinct switching regimes for smaller (*d* < 0.75*a*) and larger (*d* > 0.75*a*) antidots that is investigated in more detail for Fe antidot films by magnetometry in confined geometries, magnetic microscopy and micromagnetic simulations. Two highlights are the formation of an artificial spin-ice structure for the larger antidots and the observation of a residual out-of-plane component mediated by the smaller antidots. This transition behaviour of the switching mode directly motivates studies of out-of-plane magnetised antidot films. For perforated GdFe films we also observe a transition of switching modes, however at a smaller *d*/*a* ratio of about 0.5. FORC-MOKE investigations show magnetic switching dominated by domain wall pinning while the larger antidots reverse individually. Upon releasing the field from a fully magnetised state, we obtain stripe domains for large antidots of *d* = 165 nm at *a* = 200 nm by both, experiments and simulations. The in-plane and out-of-plane magnetised systems represent examples for the rich switching behaviour in films with periodic perforations.

## Results and Discussion

### Experimental approach and analysis techniques

In this technical section, we present the preparation of antidot lattices using nanosphere lithography and discuss important aspects of the magnetic characterisation and simulation that are not common knowledge, i.e., FORC using a MOKE microscope and the micromagnetic simulations applied to an antidot lattice.

#### Preparation of antidot lattices

Using a bottom-up approach, the preparation of antidot samples plays a crucial role and the achievements and drawbacks need a critical survey. Only with the precise control over the structural parameters, i.e., diameter and distance of the antidots, it is possible to correlate the geometry with the resulting magnetic switching modes. In nanosphere lithography, the quality of self-assembled antidot arrays is primarily determined by the size distribution of the particles, and the long-range order of their self-assembled monolayer. While top-down techniques like e-beam lithography usually require a multi-step, laborious preparation process, nanosphere lithography offers the preparation of a self-assembled monolayer on the time scale of minutes even on a centimetre length scale. In this work, polystyrene(PS)-based nanosphere lithography was chosen, since monodisperse PS nanosphere suspensions are nowadays commercially available with a wide range of sphere diameters at relatively narrow size distributions. However, the control of the self-assembly process leading to optimized samples is still challenging.

**Figure 1:**
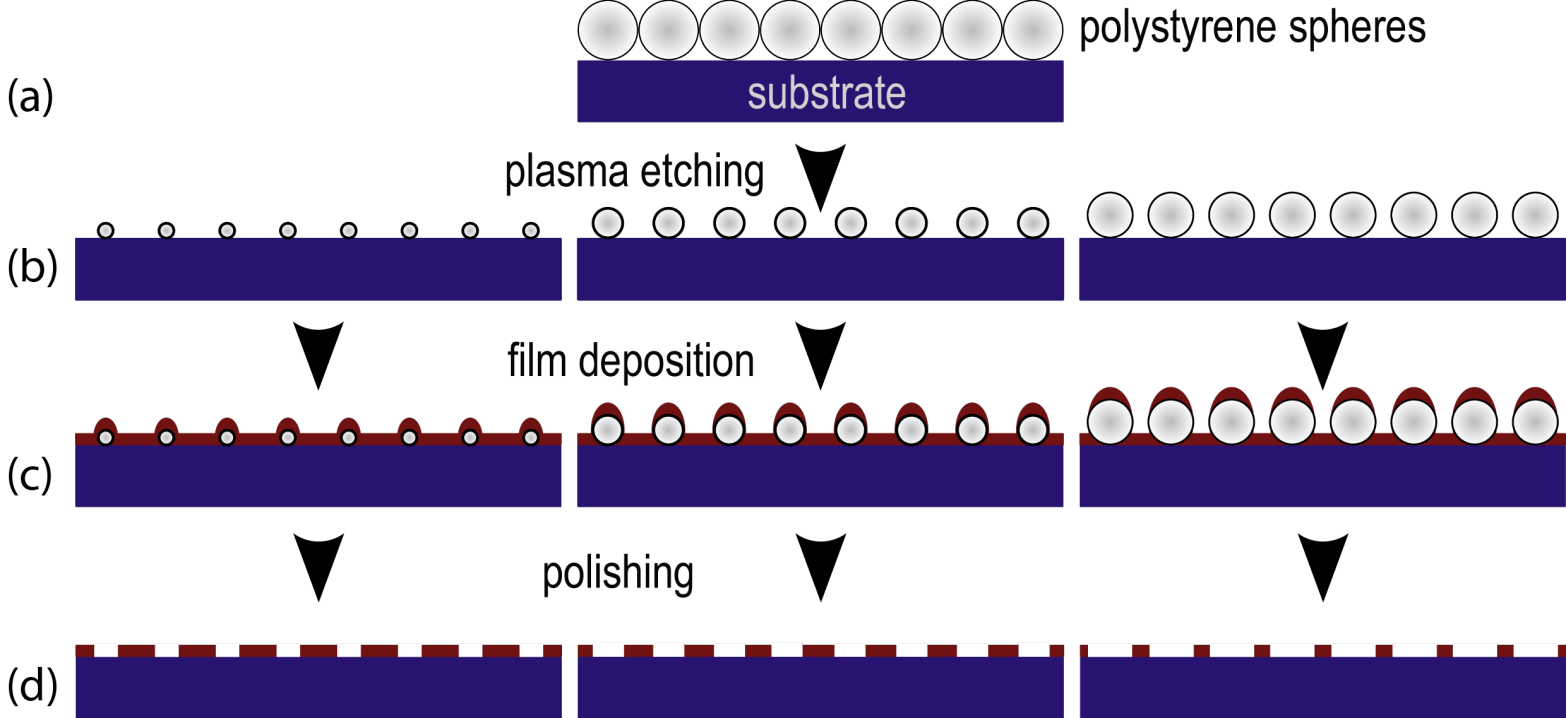
Preparation process of magnetic antidot arrays. After self-assembly of a monolayer monodisperse PS spheres (a), the spheres are homogeneously etched down to the chosen mask (b) for small, average and large antidot diameters from left to right. In (c) a magnetic film is deposited. Finally, the mask including the magnetic caps is removed by chemo-mechanical polishing (d).

Figure 1 presents the entire preparation process of magnetic antidot arrays. In order to prepare such arrays on a large scale with geometrical parameters in the 100–500 nm regime, a modified variant of colloidal lithography based on previous works [12,20] is applied. The approach exhibits remarkable flexibility as well as the possibility to pattern substrates on a centimetre scale. The preparation process consist of 4 steps: (a) the self-assembly of monodisperse PS spheres on the chosen substrate; (b) the homogeneous reduction of the PS sphere diameter by setting the plasma etching time while maintaining their initial positions on the substrate. Then, the nanoscale masks are ready for the deposition of a magnetic film and a capping layer for oxidation protection (c), and (d) the chemo-mechanical polishing for the removal of the masking spheres including their magnetic caps. In this way, we prepare hexagonal antidot lattices with antidot distances *a* = 100–500 nm at variable antidot diameter *d =* 0.1–0.9*a*. The thickness of the deposited films is limited to roughly half the antidot diameter, since the acetone-based chemo-mechanical polishing needs a sidewise access to the spheres allowing PS dissolution.

Under best conditions, we achieve defect-free antidot lattices of 25 × 25 µm^2^. Thus, integral measurements like SQUID magnetometry are always comprised of contributions from all possible antidot orientations leading to orientation averaging. As will be demonstrated below, MOKE microscopy has sufficient spatial resolution for magnetic measurements of a coherent part of the hexagonal antidot lattice, magnetic force microscopy (MFM), and scanning transmission X-ray microscopy (STXM) deliver magnetic maps even down to a single antidot unit cell.

**Figure 2:**
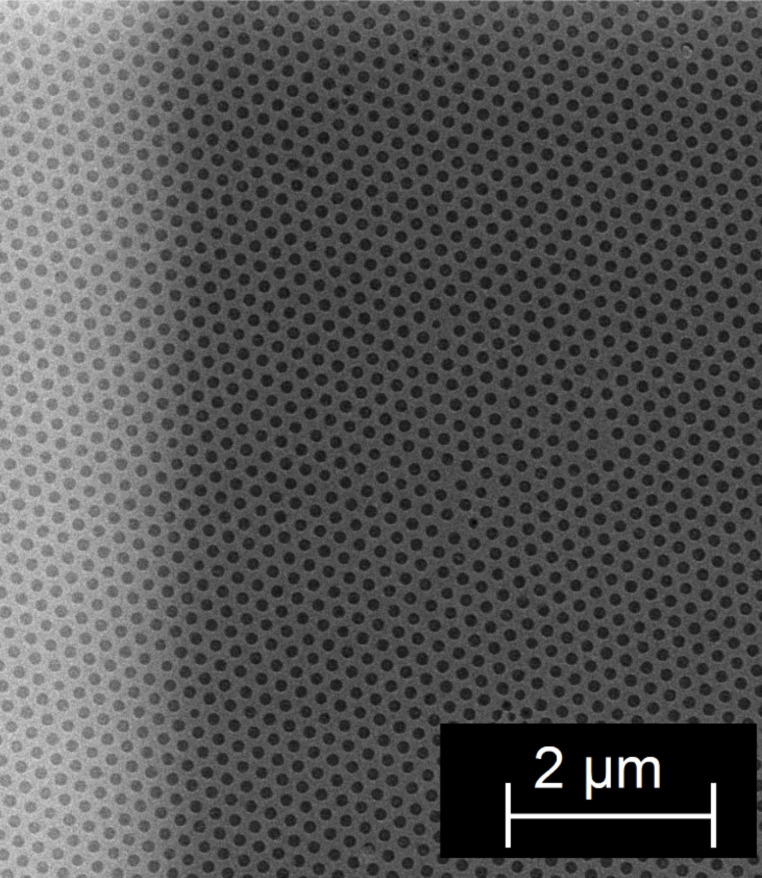
SEM image of Fe antidot array with a period *a* = 200 nm, an antidot diameter *d* = 125 nm and a thickness *t* = 20 nm. The bright contrast on the left arises from the supporting Si frame of a 500 nm thick Si_3_N_4_ membrane window (dark contrast) for transmission imaging. Remarkably, such windows survive the mechanical polishing procedure.

One example of the prepared antidot lattices is displayed in Figure 2. The scanning electron microscopy (SEM) image shows a Fe antidot array with *a* = 200 nm, *d* = 125 nm and thickness *t* = 20 nm formed on a 500 nm thick Si_3_N_4_ membrane used for STXM. The brighter contrast on the left in Figure 2 arises from the supporting Si frame of the 0.5 × 0.5 mm^2^ membrane area. Principally, the removal of PS spheres opens the door for oxidation and thus magnetic modification of the antidot films starting from the antidots’ rims. In practice, however, it turned out that neither the magnetisation of the samples nor their coercive field changed significantly over time scales of several months. The experimental parameters used for preparing the antidot samples presented below are given in the experimental section.

#### First-order reversal curve magnetometry

**Figure 3:**
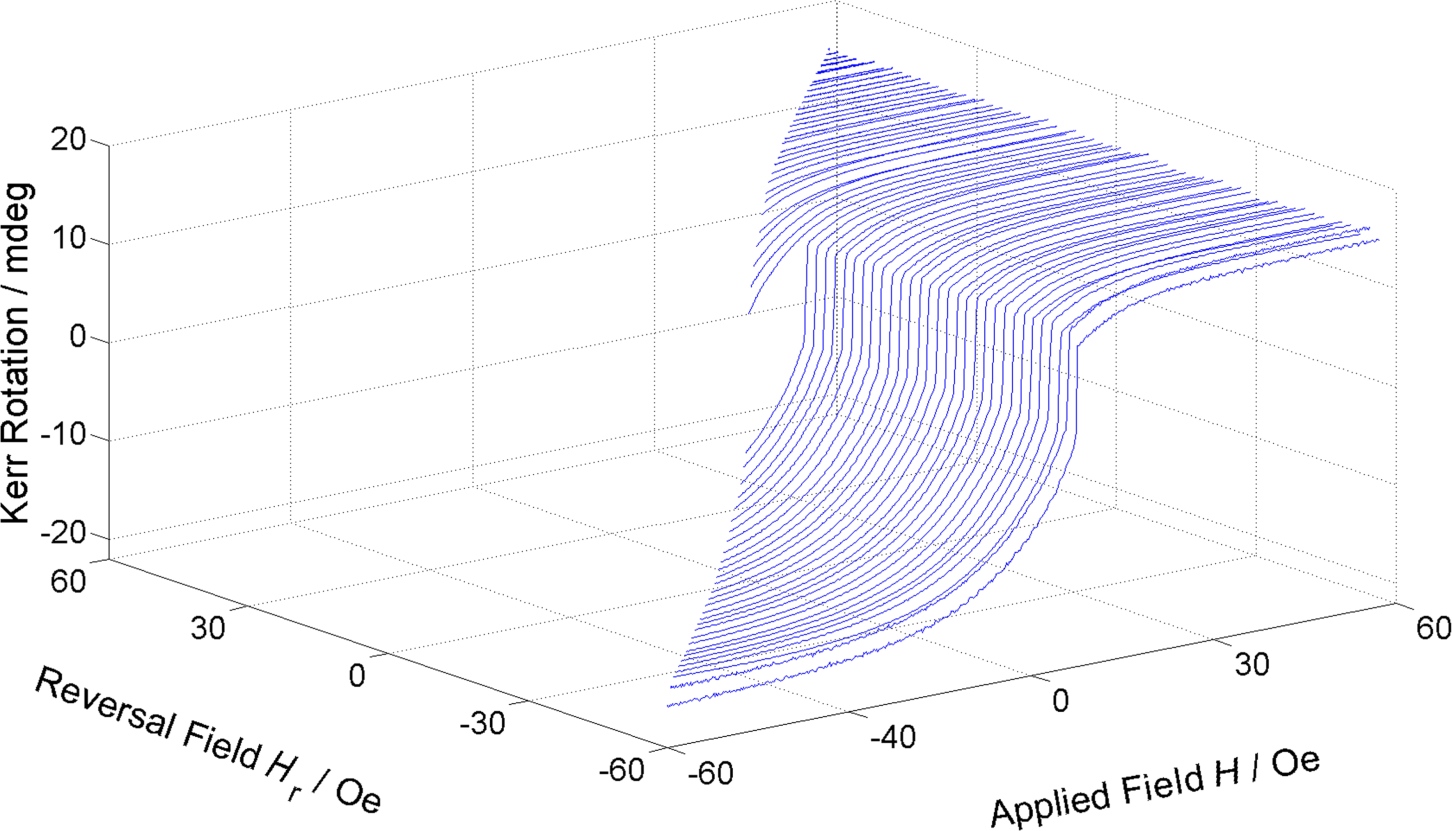
Exemplary set of minor loops for 61 reversal fields *H*_r_ with Δ*H*_r_ = 2 Oe from which the FORC density can be calculated according to Equation 1. Reproduced with permission from [23], copyright 2014 AIP Publishing LLC.

Due to the nanoscale dimension of the investigated antidot lattices, typical laboratory methods like MOKE microscopy are not able to spatially resolve the microscopic switching processes resulting in the unique magnetisation properties of these nanostructures. Hence, it is necessary to use complex and sophisticated synchrotron methods like STXM and photoemission electron microscopy (PEEM) or magnetic force microscopy. One possible way, however, gaining further microscopic understanding of interaction phenomena and coercive field distributions in complex systems is based on the well-known Preisach model [21]. Mayergoyz derived a connection to so-called first-order reversal curves (FORC) [22], which promise the ability to quantitatively extract separate individual irreversible magnetic switching events from their coercive field and interaction field. Unfortunately, FORC requires a multitude of conventional magnetometry measurements usually performed in sensitive vibrating sample magnetometers (VSM) and superconducting quantum interference devices (SQUID) by long-lasting protocols. Moreover, such setups do not provide spatial resolution, which is prerequisite for the investigation of small, spatially coherent areas in self-organized, nanopatterned antidot lattices as discussed here. Another important issue is the high field resolution necessary in the context of the presented work, which has been made available in our MOKE-FORC. This requires a large number of minor loops delivering the corresponding number of reversal fields *H*_r_. A FORC set of measurements is exemplarily shown in Figure 3, which consists of many external field *H*_ext_ dependent minor loops starting from different reversal fields *H*_r_, ranging up to the opposite saturation field.

From these minor loops, the FORC density is calculated as second order derivative according to:

[1]
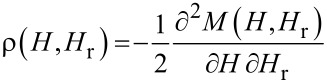


**Figure 4:**
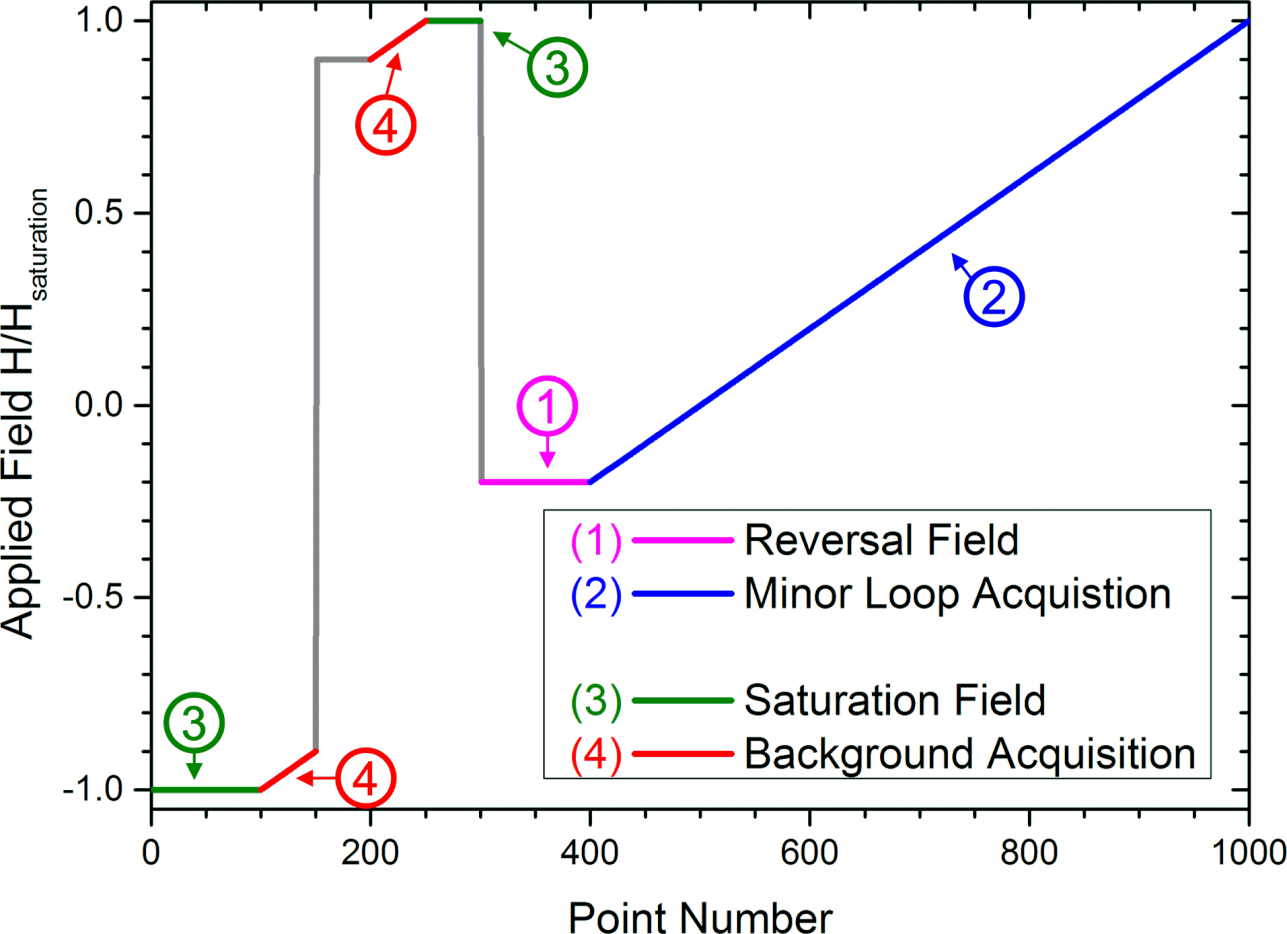
Field profile adapted to minor loop measurements with MOKE. Part 3 and 4 (green and red) are used for signal referencing. Part 1 (pink) is the variable reversal field and part 2 (blue) is related to the measurement of the minor loop itself. Reproduced with permission from [23], copyright 2014 AIP Publishing LLC.

However, measuring several hundreds of minor loops is very time-consuming using conventional magnetometers. On the other hand, faster magnetometers with possible spatial resolution based on magneto-optical Kerr effect (MOKE) strongly suffer from drift, Faraday effect background, and they do not provide absolute magnetisation values [23]. However, focused MOKE provides the necessary high spatial resolution, needed for self-organized antidot arrays as shown in Figure 2. To overcome the mentioned MOKE limitations we developed a specialized magnetic field sequence applied to the sample, as shown in Figure 4. The field sequence provides two additional anchor points at both positive and negative saturation (3) for every minor loop investigated. This allows correction for drift and normalisation to saturation values for each single minor loop. In addition, two field variations (4) are added close to the saturation anchor points, allowing consistent determination of the Faraday effect of all components in the light path (for example in the lenses nearby the magnet).

This MOKE-FORC adaption allows the fast and precise determination of FORC density maps providing a spatial detection range of 3–5 µm, i.e., much smaller than the dimensions of the structural domains of the antidot lattices under investigation (cf. Figure 2). We were able to measure one full FORC reversal curve in less than 60 seconds [23] that, in turn, enabled us to measure the magnetic switching fields of self-organized and nanostructured antidot lattice samples with very high reversal field resolution.

#### Micromagnetic simulations

Over the last years, micromagnetic simulations have proven being a most valuable tool for a detailed understanding of the static and dynamic properties of nanoscale magnets. Thanks to the increasing computational power, nowadays such simulations can predict the magnetic properties of samples with confined geometries and by mimicking the sample shape, the results can often be directly compared to the experiments. Here, we carry out micromagnetic simulations using the OOMMF package [24] for the Fe based antidot lattices and a custom-built code solving the Landau–Lifshitz–Bloch equation of motion for GdFe antidot lattices [25].

The OOMMF package rests on a continuum theory with a finite difference approach to solve partial differential equations numerically [26]. Calculations are performed by minimizing the total magnetic energy of the system, containing contributions from the exchange energy, the anisotropy energy, the demagnetizing field, and the Zeeman energy [27]. We comprise the distributions of the antidot diameter and distances by an arbitrary alteration by ±5%. The experimental constrictions along the nearest neighbour (nn) or next nearest neighbour (nnn) direction used in experiments below (cf. Figure 7) are simulated by a rectangle of 8 × 2 µm^2^ and a thickness of 20 nm using the Fe bulk material constants (*M*_S_ = 1.7 MA m^−1^, exchange constant 21 pJ m^−1^, cubic anisotropy 48 kJ m^−3^). We account for the polycrystalline Fe film by setting random anisotropy axes in the grains of 10 × 10 × 20 nm^3^. The angle of the magnetisation vectors of neighbouring cells is controlled by a reasonable small mesh size (2 × 2 × 10 nm^3^) [28].

As will be shown later in connection with the anisotropic magnetoresistance (AMR) the major part of the total resistance arises from the 2.5 × 20 µm^2^ FIB-cut constriction. But still the continuous antidot film surrounding the channel influences the magnetic switching via dipolar interactions. In our simulations, the elongated sample size (2 × 8 µm^2^) results in anisotropy along the long axis while we do not observe any shape anisotropy of the channel at an aspect ratio of 8 in our experiment. To account for this discrepancy we have modified our simulations by applying an additional uniaxial anisotropy with same axis but opposite value of the shape anisotropy. Using this procedure, the shape anisotropy is cancelled out and our simulations are closer to the experiments.

During hysteresis, domain nucleation will turn out to be an important factor affecting the reversal process [27]. As the nucleation often happens at the edge of a sample we take care of the peculiarities of the edges: First, the simulated long edges (8 µm long) are non-perfect with a certain amount of roughness. Second, the upper edge is placed roughly in the middle of the holes, while we positioned the lower one roughly between two rows of antidots (cf. sample geometry in Figure 7). Thus, many different nucleation scenarios are potentially possible and we only use the centre square of 2 × 2 µm^2^ of the simulated structure allowing for them. This approach is described in more detail elsewhere [29].

To calculate the AMR signal from the simulated magnetisation we perform the following procedure [29]: First, the inhomogeneous current density is calculated considering both, the complex sample structure and the magnetisation pattern. Then, the total AMR signal is obtained by integrating the current density. For calculating the current density, we use the software package IDC2D [30]. We point out that the chosen method is not the only one possible: for instance, instead of the current density one could evaluate the local electric field. We have tested different approaches, concluding that the differences between them are small and influencing mainly the size of the AMR peaks but not their positions. Details of these tests are thoroughly discussed in [31].

For simulations of thin GdFe films with perpendicular anisotropy, a different micromagnetic code is used. It is based on the improved version of the Landau–Lifshitz–Bloch (LLB) equation of motion [32]. The code successfully solves the µMAG standard problems 3 and 4 [33]. In another test, a complex domain structure in a thin film is compared with that obtained from a well-established LLB code, showing good agreement. The implemented multi macrospin model involves only one sublattice. This is obviously a drawback concerning simulations of ferrimagnetic materials. However, up to the present day, there is no theoretical multi macrospin model for two sublattices solving the LLB equation and we used the existing one-sublattice model instead. This is a proper approximation as long as the antiferromagnetic coupling between the two sublattices of the simulated material is stronger than other effects. In this case, the magnetic moments of the two sublattices can be locally summarized to one magnetic moment (e.g., one macrospin). The LLB equation requires several material parameters and temperature dependent input functions. The latter are the exchange stiffness, the equilibrium magnetisation, and the parallel and perpendicular susceptibilities. The parallel susceptibility can be related to the uniaxial anisotropy [34–35]. In the past, these input functions were obtained from atomistic simulations (e.g., based on the Landau–Lifshitz–Gilbert equation of motion) for the special case of FePt [25,34]. Here, these functions have been rescaled to fit the properties of GdFe. To do so, one needs to know the Curie temperature, the saturation magnetisation, the uniaxial anisotropy and the exchange stiffness at 0 K for the particular material. A realistic value for the micromagnetic damping constant is in the range of 0.1 [36–37]. In case one is not interested in the dynamics but only in the equilibrium state of the system, the damping can be increased to 1.

### Magnetic switching of in-plane magnetized antidot films

#### Integral magnetic properties

**Figure 5:**
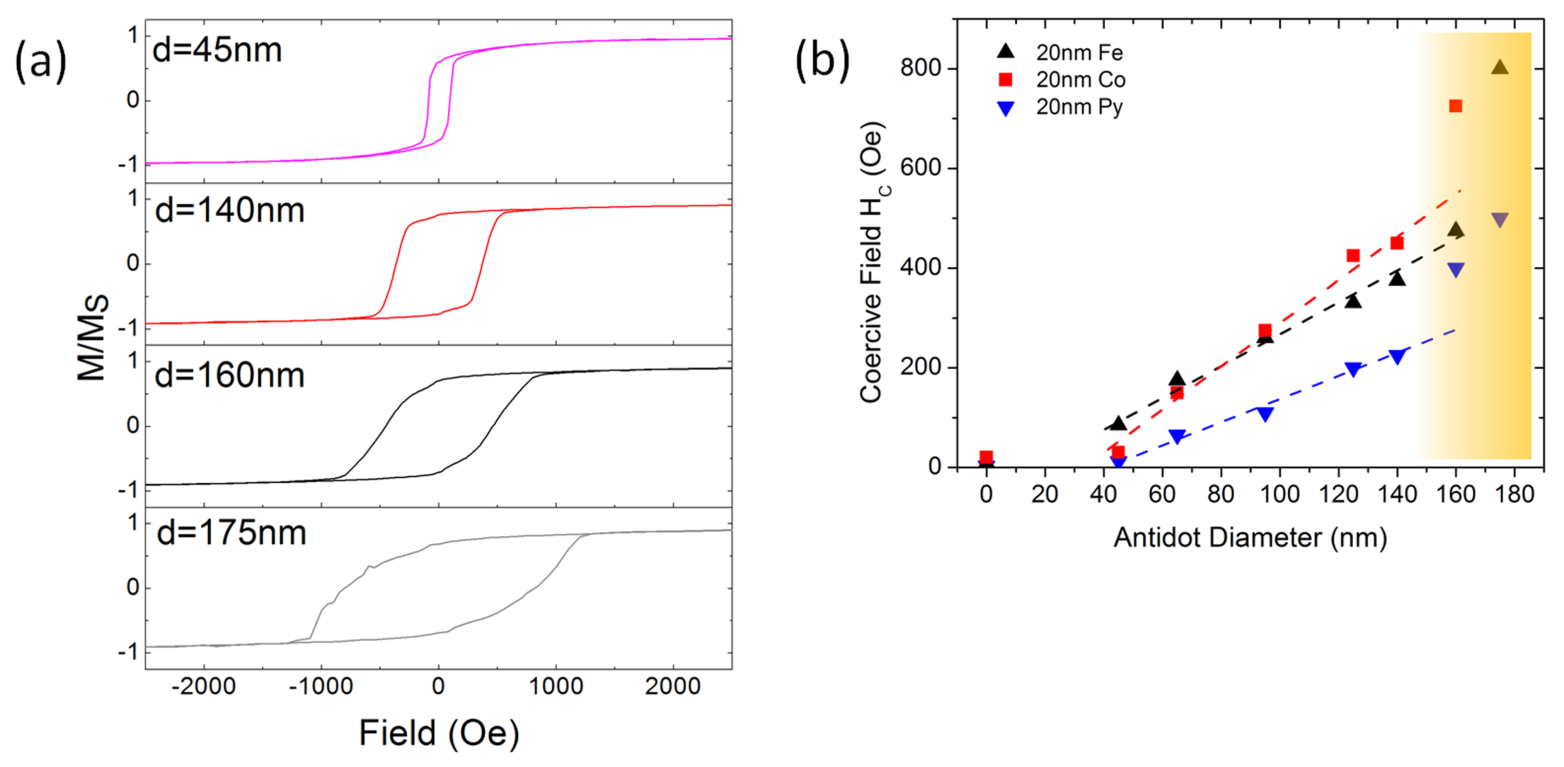
(a) In-plane hysteresis loops of 20 nm thick Fe antidot arrays with constant period of *a* = 200 nm and varied antidot diameter *d* measured at *T* = 300 K. The coercive field rises monotonously with increasing antidot diameter. The hysteresis loops change from a square-like behaviour for smaller antidot diameters *d* < 150 nm towards a more convex shape with a much wider switching field distribution for larger diameters *d* > 150 nm. Panel (b) presents the coercive field of Fe, Co, and Py antidot films as function of *d*. All materials show a linear increase of the coercive field between *d* = 45 nm and *d* = 150 nm as indicated by the dashed lines. For larger antidots, the coercive field jumps to significantly larger values as indicated by the orange shaded region.

In this section, we present the magnetic hysteresis loops of Fe, Co, and Py antidot films of varying antidot diameter *d* as determined by SQUID magnetometry. These integral results – averaged over the thousands of structural domains of the antidot lattice – serve as a starting point for the following discussion of microscopic switching mechanisms obtained from magnetic microscopy techniques and micromagnetic simulations. Figure 5a presents the normalized magnetic hysteresis loops of Fe antidot films with *d* = 45 nm, 140 nm, 160 nm, and 175 nm at a constant period *a* = 200 nm and temperature *T* = 300 K. Note that for all materials antidot films are grown in a single deposition process. In this way, we can guarantee identical deposition conditions and a constant thickness of *t* = 20 nm. We obtain a drastic increase of the coercive field from about 10 Oe (*d* = 0 nm, not shown) to 95 Oe for *d* = 45 nm while the hysteresis still maintains a rather rectangular shape. Further increase of the antidot diameter leads to a linear increase of the coercive field as shown in Figure 5b for Fe, Co, and Py antidot films. Remarkably, this finding is in agreement with previously published results for antidots arranged in square lattices prepared by conventional lithographic techniques [38–39] and underlines the suitability of the self-assembly method. Further, geometry dependent coercive fields have also been discussed in the work of Smyth et al. [40]. There, the coercive field is tuned by the aspect ratio of ferromagnetic nano-bars. They found a monotonous increase with the bar’s aspect ratio due to increasing shape anisotropy. As will be shown in the next section by means of magnetic force microscopy and micromagnetic simulations, the remaining ferromagnetic material between two neighbouring holes can (under certain circumstances) be treated as a single domain bar magnet, and a larger antidot diameter increases the aspect ratio of these bars. This suggests that shape anisotropy is an important driver for the augmented coercive field in the antidot arrays.

The Fe antidot array’s hysteresis loop in Figure 5 can be interpreted in a way that upon applying a small reversal field, an additional anisotropy caused by the nanostructures holds the local magnetisation in its original direction and only a small slope of the magnetisation curve is observed. This clearly indicates a reversible rotation process. Once this anisotropy is overcome, the susceptibility increases drastically, suggesting that the reversal process is driven by nucleation and growth of reversed domains, i.e., irreversible processes. This movement is hindered by domain wall pinning (by the antidots), as the reversal is not complete until a further increase of the counter field is applied.

Further changes of the magnetisation can still be observed up to fields of 8000 Oe, while the reference film saturates at a field of 45 Oe (not shown). This can be regarded as an additional support for the hypothesis that shape anisotropy plays a significant role in the alteration of the magnetic properties: it requires rather large fields to force the magnetisation to point into the holes. While for diameters below *d* = 140 nm the loops are more rectangular, above this diameter the loops become more and more distorted towards a convex shape with a much broader switching field distribution. This finding gives a first hint towards a change in reversal mechanism when the antidot diameter is increased above the threshold of *d* > 0.75*a*, observed for Fe, Co, and Py antidot films (cf. Figure 5b). All materials show very similar trends. It is noticeable that Py exhibits the smallest coercive fields for all antidot diameters, while the coercive fields for Iron and Cobalt are approximately on the same level for antidot diameters below *d* = 95 nm. For larger antidots, the coercive field of the Co antidot arrays exceeds the values obtained for Fe.

At this point, the question arises how various material parameters influence the magnetisation reversal. The materials differ in their saturation magnetisation *M*_S_, with Fe (1700 kA m^−1^) displaying the highest value followed by Co (1430 kA m^−1^) and Py (800 kA m^−1^). On the other hand, the Co exchange constant *A* = 30 pJ m^−1^ is more than twice the one of Py (13 pJ m^−1^), while the one for Fe (21 pJ m^−1^) lies in between the other two [41]. A closer look at the magnetisation reversal for the three different materials delivers a reasonable first interpretation from the integral hysteresis loops [41]. The shape anisotropy introduced by the antidots balances with the Zeeman energy generated by the external field, and upon further increase of the field the reversal happens via domain wall movement. On the other hand, the local shape anisotropy is based on interaction of the stray field (generated by its magnetisation) with its own magnetisation 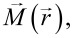 thus the resulting anisotropy energy should increase with increasing *M*_S_. However, the effect of the growing interaction of the external field with a higher saturation magnetisation is superimposed, which depends on the exact sample magnetisation 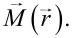 This problem is too complex for an analytical solution. The micromagnetic simulations below will help elucidating the exact reversal mechanisms.

Co, the material with the largest exchange constant, shows the flattest curve and, thus, has the broadest switching field distribution. The relatively small slope of the *M*(*H*) curve points towards a strong domain wall pinning effect. According to Paul [42], the energy of a domain wall is proportional to its spatial dimensions and the exchange constant *A* of the magnetic material, as well as to the anisotropy constant *K*. Among the three materials under investigation, the domain wall energy in Co antidot films should be the highest. In a very basic model, one can attribute the pinning effect simply to the fact that a domain wall, which incorporates a non-magnetic hole, reduces its energy because it minimizes its volume, in analogy to the domain wall pinning in a ferromagnetic nanowire containing a notch [43]. In turn, this pinning becomes more effective the more energy is saved by incorporating the non-magnetic defect into the domain wall, i.e., it scales with the domain wall energy. In this way, the high depinning fields, which are necessary to complete the magnetisation reversal for the Co antidot array, are qualitatively explained.

#### Magnetic microscopy and micromagnetic simulations

The integral magnetometry data presented above suggest complex magnetic reversal mechanisms with a change of the switching modes at a ratio of the antidot diameter *d* to the antidot period *a* of about 0.75. Due to orientation averaging, however, integral techniques cannot provide any information on the in-plane anisotropies imprinted by the hexagonal antidot lattice. Thus, more information from structurally perfect antidot domains is needed which can be provided by magnetic microscopy techniques or transport measurements in confined, well-oriented geometries.

#### Antidot lattices with *d*/*a* ratio >0.75: spin ice configuration

**Figure 6:**
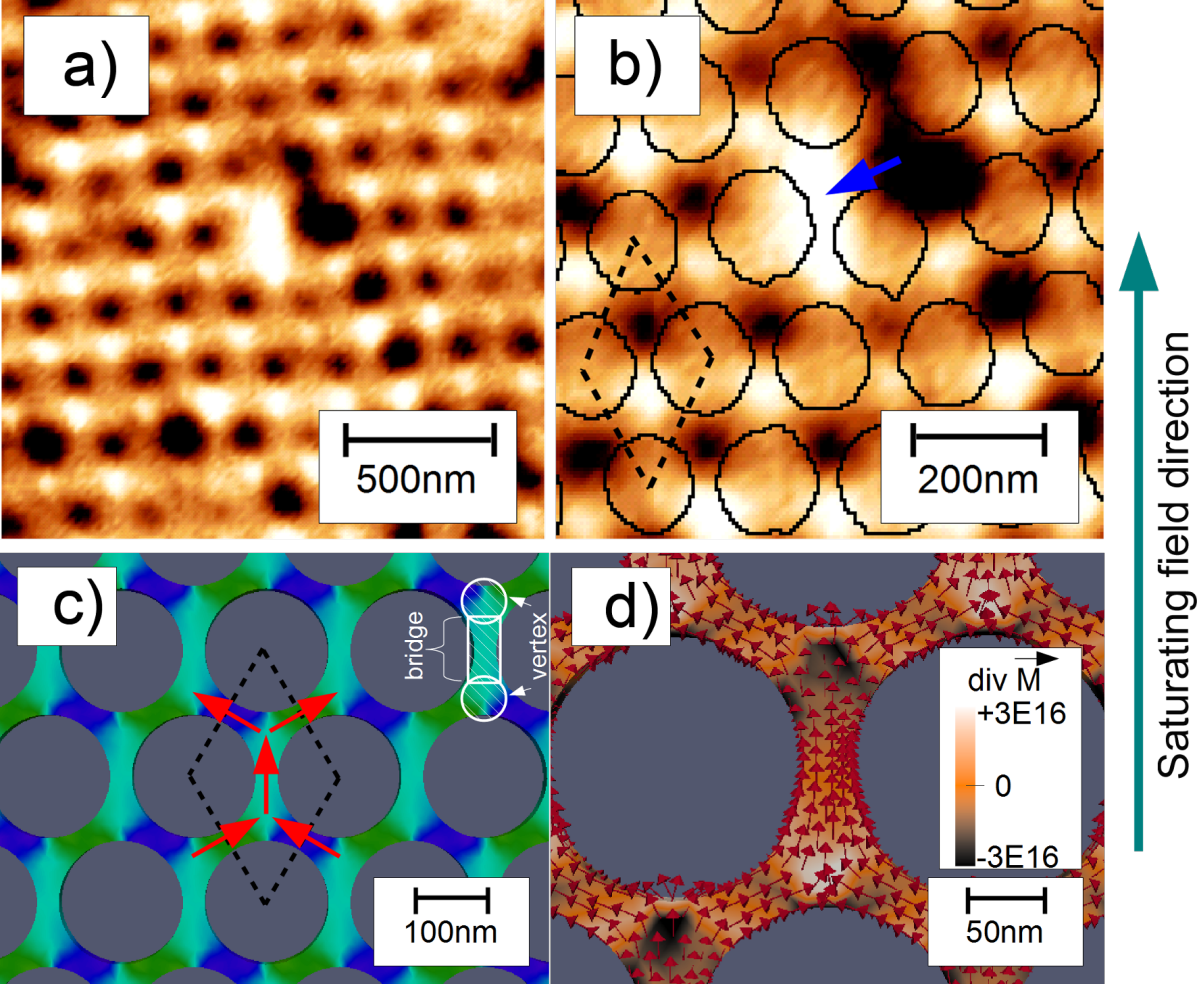
Domain pattern of hexagonal Fe antidot arrays with lattice parameter *a* = 200 nm and hole diameter *d* = 170 nm in the remnant state after saturation in an external field. (a) shows an 1.5 × 1.5 µm^2^ MFM image while the positions of antidots as observed by AFM topography-scans and software recognition are added to panel (b) in a magnified region of (a). The tip–sample distance was set to 10 nm. In (b), the blue arrow indicates a single flipped bridge magnetisation. Panel (c) presents the simulated magnetisation pattern for the experimental antidot geometries. Red arrows indicate the direction of magnetisation in each domain of the unit cell (dashed lines). In panel (d) the colour code represents the magnetic charge distribution 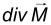 derived from the magnetisation vectors (red arrows). Reproduced with permission from [16], copyright 2013 IOP Publishing Limited.

When the PS masks are only slightly etched, i.e., for large antidot diameters, a significantly different switching behaviour is observed as compared to the antidots with smaller holes. The magnetic material forming the antidot structure has an elongated, waisted shape along two neighbouring holes and vertices arise in the centre, in between three neighbouring holes. At the vertices, three of these structures are connected to each other, thus exhibiting exchange coupling. According to their function of “bridging” two neighbouring vertices and due to their elongated shape, these structures are labelled “bridges”. In Figure 6a and Figure 6b we obtain alternating bright and dark contrasts in a honeycomb structure for an antidot diameter of *d* = 170 nm. MFM detects the stray fields, and these bright and dark spots are located at the vertices of the bridges surrounded by three antidots. The bridges have an elongated shape, and thus, the local magnetisation points towards the long axis. Since three bridges have a common junction, a residual magnetic charge can be defined, which is the source of the magnetic stray field resulting in the contrast in the MFM investigations [16].

This interpretation is supported by the micromagnetic modelling of the system. Figure 6c shows the simulated magnetisation pattern while Figure 6d presents the magnetic charge distribution 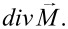 As indicated in Figure 6c, the primitive magnetic unit cell consists of five domains in accordance to the five bridges, which are at least partially implied by the unit cell. The magnetisation of the central domain magnetisation points towards the next nearest neighbour direction closest to the direction of the initially applied magnetic field, while the other four domains have their magnetisation oriented to the next nearest neighbour directions enclosing angles of 60° with the magnetisation of the central bridge magnetisation. Consequently, the periodic domain pattern is only formed when the field is not applied to one of the nearest neighbour directions of the antidot lattice, as this would result in the field pointing perpendicular to the long axis of a bridge. Upon switching off the external field, the direction into which the bridge’s magnetisation then relaxes would be random or determined by defects in the lattice, which makes the occurrence of a periodic state highly unlikely. The influence of defects on the magnetisation pattern is also exemplified in Figure 6b. The magnetisation of the bridge marked by the blue arrow is switched due to misaligned adjacent antidots, breaking the alternating pattern.

The arising periodic structure of bright (positive) and dark (repulsive) interactions with the MFM tip is described best using magnetic charges as suggested before [44]. We assign the charges ±*q*_i_ = µ/*l* with µ being the magnetic moment of a bridge and *l* its length to each end of the *i*-th bridge, respectively. With three bridges being connected to each vertex, there are a total of four possible charge states a vertex can attain: *q*_vertex_ = ±*q* or *q*_vertex_ = ±3*q*. The ±3*q* states would result in a three times higher signal than the ±*q* states in the MFM images [45]. These states, however, are not observed for the antidot lattices under investigation. The ±*q* vertex states result from two connected bridges having a magnetisation direction towards the vertex, while the third has a magnetisation pointing away from the vertex, or vice versa. These are the well-known ice-rules for a two dimensional Kagomé spin-ice [46–47]. In fact, the experimentally observed magnetisation state in which each dark (−*q*) vertex has three neighbouring bright (+*q*) vertices (and vice versa) is exactly the so-called charge ordered state of such an artificial spin-ice structure. This state is supposedly very stable, as it not only minimizes the energy of each single vertex in conformance with the ice rules, but also the interaction energy between neighbouring vertices due to the alternating arrangement of neighbouring vertex charges. It often serves as starting point for subsequent magnetisation reversal experiments to investigate frustration phenomena in such model systems [48–49]. Interesting magnetisation reversal phenomena have so far been found in this kind of systems, such as the occurrence of so-called “monopole defects” [50], which essentially break down to violations of the ice rules, as well as a magnetisation reversal along a one-dimensional path [17].

The preparation of artificial spin-ice structures by means of nanosphere lithography offers additional possibilities as compared to established e-beam lithography. With its easy scalability, the technique allows to reduce the periodicity way below previous limits of about 500 nm. As the anisotropy energy, and thus the stability of the magnetisation, scales with the volume of the elements assembling the spin-ice lattice, one could imagine accessing dimensions in which the magnetisation can be activated thermally. So far, this was only possible by reducing the thickness of the magnetic material below 3 nm [51]. Recently it was shown that Py antidot lattices with periodicities of 500–3000 nm and an additional thickness modulation can be used to stabilise artificial vortex structures in the vertices [15]. Neighbouring vortices are interacting via the thinner connecting bridges leading eventually to vortex-antivortex magnetic configurations where the antivortex is located at the bridge. Even one step further, an artiﬁcial skyrmionic lattice has been prepared using a heterostructure of a Py film – (Co/Pd) multilayer with in-plane and out-of-plane anisotropy, respectively. Such vortex or skyrmion structures are probably not observed in the present experiments due to the small structure size with vertex diameters about 50 nm and bridge width of about 30 nm.

#### Antidot lattices with *d*/*a* ratio <0.75

The intrinsic domain structure in antidot arrays always stands in close relation to the orientation of the antidot lattice. According to the hexagonal lattice of holes, we expect a 6-fold in-plane anisotropy. In this section, we investigate the anisotropic switching for a ratio *d/a* ≈ 0.5, i.e., well below the above identified ratio for a modified switching mode. As stated before, an in-plane anisotropy is not resolvable by standard magnetometry as the self-assembly is limited to well-oriented antidot lattices of 25 × 25 µm^2^ and thus, we average over all directions in the film plane in a 5 × 5 mm^2^ sample used for SQUID magnetometry or magnetotransport measurements.

**Figure 7:**
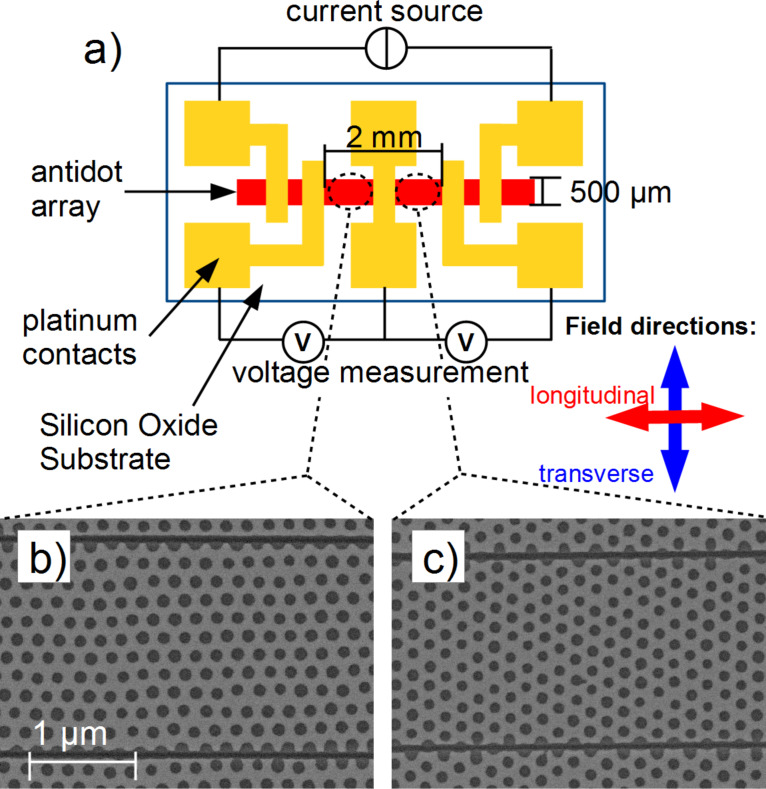
(a) Schematics of the sample geometry for AMR measurements. The red and blue arrows indicate the direction of the magnetic field for longitudinal and transverse geometries, respectively. SEM images show antidot arrays with FIB-milled cuts (dark lines) along the nearest neighbour (b) and along the next nearest neighbour direction (c). Reproduced with permission from [29], copyright 2013 IOP Publishing Limited.

However, slight modifications by FIB cutting offers an easy way to address the anisotropic in-plane properties by means of magnetotransport measurements. This is achieved by channelling the electrical current through a narrow constriction, which only extends over a single crystalline domain. For this purpose, we prepare a magnetic antidot bar on a SiO_2_ substrate as indicated in Figure 7a. The antidot array has a Fe film thickness of *t* = 20 nm, lattice parameter of *a* = 200 nm and an antidot diameter of *d* = 100 nm. The two outer contacts serve as electrodes for a maximum probing current of 10 µA to prevent any damage. The used geometry allows conducting two independent four-point resistance measurements simultaneously [29].

Within each half of the antidot array, an antidot crystallite of sufficient size and suitable orientation is found by SEM imaging integrated in the FIB device (Zeiss NVision 40 SEM/FIB). The size of the chosen crystallite is critical as it directly determines the length of the FIB milled channel, and thus, the ratio between the electrical resistance originating from this channel and the feeding contacts. In order to create a local probe for the magnetic switching, this ratio should be as high as possible. The criterion for the crystallites’ orientation is that one of the outstanding antidot crystal axis, i.e., either nearest neighbour (nn) or next nearest neighbour (nnn) direction, is aligned parallel to the net current direction. The approximately 60 nm wide and 20 µm long FIB cuttings were performed using Ga^+^ ions with a current of 10 nA for a exposure time of 9 minutes, each. Figure 7b and Figur 7c show the resulting FIB channels with the current applied (b) along the nn-direction and (c) along the nnn-direction. Both channels have a width of about 2.5 µm and a length of 20 µm, thus containing more than 1000 well ordered antidots. We obtain a 6-fold increase of the resistance for the FIB-cut samples meaning that the AMR signal mainly arises from these constrictions.

**Figure 8:**
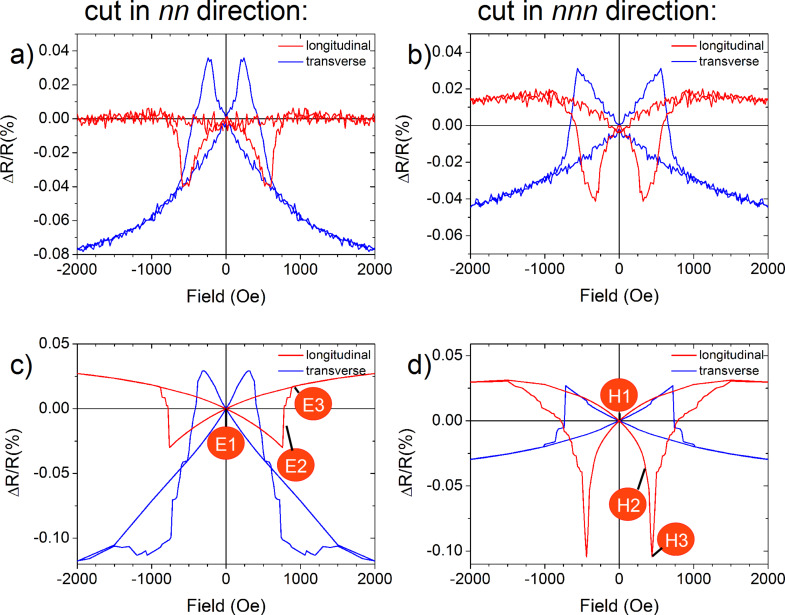
Measured AMR curves (a) for the current direction along nearest neighbours (nn) and (b) next nearest neighbour directions (nnn) in longitudinal (red) and transverse (blue) geometry of the external field at *T* = 77 K. Panels (c) and (d) present micromagnetic simulations of the corresponding geometries in identical colour code. The red indicators in panels (c) and (d) mark significant points of AMR curves for easy (E) and hard axis (H) magnetic switching. Reproduced with permission from [29], copyright 2013 IOP Publishing Limited.

In the longitudinal and transverse AMR curves, we observe dips and peaks for the channels cut in nn- and nnn-directions at *T* = 77 K (cf. Figure 8a and Figure 8b). The measurements are normalized to the remnant state magneto-resistance *R*(*H* = 0). The longitudinal geometry (red) yields dips while peaks are present in the transverse geometry (blue) matching earlier experiments [52–53]. In AMR experiments on continuous films, we have shown that peaks and dips are a good measure of the coercive field [41]. Thus, the coercive field varies significantly with the field direction allowing for measurements of the antidots’ in-plane anisotropy [29]. As shown in Figure 8a for instance, dips are present at 570 Oe for the longitudinal configuration while peaks are found at 240 Oe for the transverse geometry in the nn-direction. These two different coercive fields are clearly associated to the angle of the external field and the antidot orientation and we identify the longitudinal geometry probing one easy axis, as the coercive is largest in the nn-direction.

On the other hand, the transverse geometry consequently means that the field is applied along the nnn-direction due to the hexagonal symmetry of antidots. With its lower coercive field, this direction is the hard axis of magnetisation. Here, we can rule out that the shift of the coercive fields arises from the macroscopic rectangular shape of the channel, since we find the higher coercive field (580 Oe) for the transverse AMR-measurement in nnn-direction (Figure 8b) that is again along a nn-direction. Additional angular-resolved MOKE investigations on these channel geometries reveal a 6-fold symmetry of the coercive field (not shown).

The micromagnetic simulations directly adopted to the experimental geometries are presented in Figure 8c and Figure 8d. We obtain striking analogies with the experimental AMR curves. For all four geometries, the overall shape, the relative positions and magnitudes of the peaks and dips are in good agreement, while the absolute values in the simulations are about 20% larger as compared to the experiments. We ascribe this observation to the temperature of *T* = 77 K in the experiments while the micromagnetic simulations suppose *T* = 0 K.

The evaluation of the transverse AMR measurements can be discussed accordingly [29]. The micromagnetic simulations shown in Figure 8, panels c) and d) provide two distinct reversal modes of the magnetisation, one for the nn-direction (easy axis) and another for the nnn-direction (hard axis). Some particular points of the simulated AMR curves in Figure 8c and Figure 8d are indexed by “E” and “H” for easy and hard axis magnetic switching, respectively.

We start the discussion with the easy axis reversal (nn-direction). From AMR (Figure 8), MFM [16], and corresponding micromagnetic simulations we identify the remnant state (E1) consisting of a periodic magnetisation pattern with 5 domains. One larger central domain oriented in nn-direction is surrounded by four smaller domains enclosing an angle of 30° with respect to the initially applied magnetising field [54–55]. From the micromagnetic simulations, we derive histograms of angular distribution of the cell magnetisations. Three distinct directions of maximum weight arise at 150°, 180° and 210° [29]. The simulated magnetic domain pattern is stable indicated by its high coercive field and produces a high resistance state in the AMR. The latter is reasonable, since the generic AMR relation *R* = *R*_0_ + Δ*R* cos^2^(θ) with θ being the angle between the current and the external field direction suggests *R* = *R*_0_ + Δ*R* in the saturated state. Consequently, we observe the highest magneto-resistance in the remnant state, and the decreasing resistance with increasing external fields. One may note that these variations are reversible at larger external fields. When the external field is set to zero, the sample reaches the E1 state. When a reversal field is applied, the domain structure is almost stable up to the state E2, after which the irreversible switching processes start. Detailed MFM studies and simulations have shown that reversed mesoscopic domains have nucleated and grown [29]. After magnetic switching, we find a reversed, but again periodic domain structure in the state E3. When the external field is further increased, the four side domains gradually rotate towards the field direction until reaching the saturated state.

The hard-axis reversal is measured in the nnn-direction. As opposed to the nn-direction, the magnetisation first relaxes towards a nearby easy axis in the state H1 after releasing the external field. In this case, however, the obtained magnetisation pattern is no longer collinear to the external field, but rotated by 30° from the external field direction. When the reversal field is gradually increased, the magnetisation is pulled towards its direction (H2). In state H3 the coercive field is reached, and the experimental and simulated observation is that the magnetisation pattern intersects into many mesoscopic domains. Locally, all these domains are oriented along or close to one easy axis direction. Again, the angular distribution histograms derived from the simulations reveal three maxima at about 65°, 100°, and 130° (not shown). Thus, the major part of the total magnetisation is directed towards an easy axis configuration at 90°, slightly distorted to higher angles by the reversing field. This state produces the largest angles of the current density and the magnetisation, and thus, the AMR exhibits a minimum resistance state [29]. At larger external fields the magnetisation vector turn into the next easy axis direction at 30°, before the saturated state is reached for larger reversal fields. Overall, we obtain a sequenced population of neighbouring easy axis, starting from the 150° configuration, via the 90° easy axis, and towards the 30° angle between the magnetisation and the external field. Such switching mode was also observed by Manzin et al. [56]. In this work, hexagonal Py antidots were simulated using periodic boundary conditions as opposed to the present contribution. This idealisation, however, results in a collective turning mode without any mesoscopic domains, which is in contradiction to the experimental findings. This magnetic reversal via distinct intermediate easy axes is also measurable by MOKE-microscopy, directly applied to the AMR samples in Figure 7. See [29] and [57] for details.

**Figure 9:**
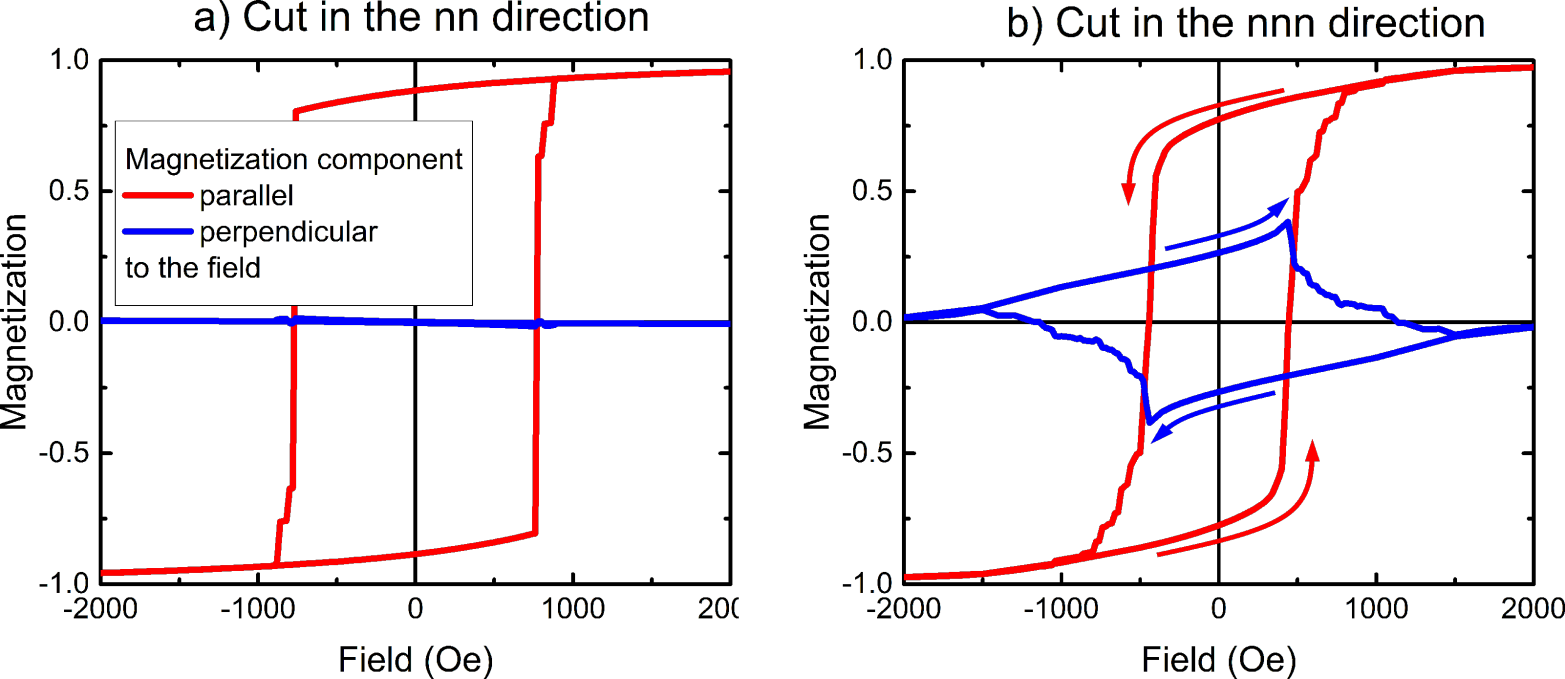
Micromagnetic simulation of hysteresis curves corresponding to the AMR measurements. Hysteresis of a 2 × 2 µm^2^ sample piece is calculated for the magnetic field applied along the nn-direction (a) and along the nnn-direction (b). Magnetisation component along the field (usually shown in hysteresis figures) is marked in red. Orthogonal component (in-plane of the antidot matrix) is shown in blue. Panel b) reproduced with permission from [29], copyright 2013 IOP Publishing Limited.

**Figure 10:**
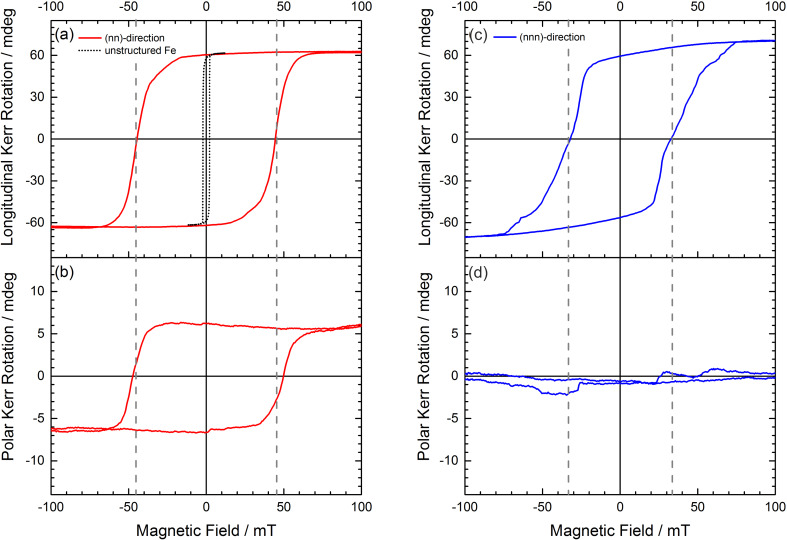
(a) Longitudinal and (b) polar Kerr hysteresis loops with an in-plane magnetic field applied along the nn-direction of a hexagonal antidot lattice with *a* = 200 nm antidot periodicity and *d* = 100 nm antidot diameter in a *t* = 20 nm thick Fe film. For comparison, the hysteresis loop of the unstructured film is additionally shown as dotted curve in (a). Panels (c) and (d) present the longitudinal and polar Kerr hysteresis loops with an in-plane magnetic field applied along the nnn-direction, respectively. The right panel has been reproduced with permission from [57] under CC-BY 3.0 license, copyright 2015 IOP Publishing Limited.

Here, we compare and explain the results from micromagnetic simulations (Figure 9) and MOKE microscopy (Figure 10). Both approaches reveal that the hexagonal antidot lattice possesses a 6-fold symmetry. In such a lattice the nn-direction is an easy axis, while the nnn-direction is a hard axis [31]. We point out that this statement should be interpreted with some precaution. If the system is stabilized along one of its easy axes, not all magnetic moments point exactly in this direction as shown in [29]. The sample as a whole is indeed magnetised in this direction, but locally the spins are distributed around three directions: at about 150°, at 180° and roughly at 210°. This is due to dipolar interactions and known as the pole-avoidance principle [27]. According to this rule the magnetisation pattern tries to avoid volume charges (in that particular case absent) and surface charges – i.e., the magnetisation tries to align parallel to the sample edge. As a result many small domains are formed each of them pointing in either of three directions mentioned above. They all form, however, one mesoscopic domain pointing on average in the direction of the 180° easy axis. This explanation was further confirmed by MFM measurements [29].

The knowledge of the hexagonal anisotropy allows us to explain the magnetisation reversal process during the AMR measurements. First, we discuss the case of switching in nn-longitudinal and nnn-transverse systems. Here, the magnetisation is reversed along the easy axis having the larger coercive field (Figure 9a). Secondly, during the switching a domain appears that is parallel to the applied field (antiparallel to the initial field direction). As a result, no domains orthogonal to the field are present during switching. Please recall that the AMR signal is proportional to cos^2^(θ), where θ is the angle between the current and the magnetisation. Thus, during switching, θ remains either close to 0° or to 180° (for nn-longitudinal) and to 90° or to 270° (in case of nnn-transverse). This behaviour leads to small changes in the AMR signal and the dips/peaks are small. The last property of the AMR curve is related to the process of nucleation and propagation of domains. Namely, micromagnetic simulations show that in the present case, this process is fast, i.e., the hysteresis is rather rectangular. As a result, the AMR dips and peaks are narrow.

The opposite situation occurs during the hard axis reversal (nn-transverse or nnn-longitudinal). Now, the hysteresis is narrower – for obvious reason (cf. Figure 9b). This means that the AMR features are closer to each other. Secondly, during the switching there are some easy axis states available for the system, states that are a favourable alternative to hard axis (thus high energy) initial- and final states. Because of these intermediate states, the hysteresis curve is now smoother and less rectangular and each of the AMR features is now wider compared to the previous case. Lastly, the magnetisation in these intermediate states points into easy axis directions being at angles of 60° (or 120°) to the field direction. In case of an ideal sample, these contributions would cancel out – the magnetisation component that is orthogonal to the field would be zero. In reality, however, the sample is not perfect, and we consider this fact in our simulations as well [29]. As a result, the perpendicular magnetisation component is non-zero (cf. Figure 9b). This phenomenon is also experimentally confirmed by MOKE microscopy (Figure 10) and MFM observations [29].

As has been discussed above in the context of artificial spin ice structures, the hexagonal antidot lattices produce a geometric frustration of the magnetisation at the vertices. However, this frustration is not limited to the two dimensional formation of pairs of magnetic monopoles or the in-plane spin ice structures discussed above. Significant tilting or bending of the magnetisation around the antidots could be present, providing an extension to the third dimension in these initially in-plane magnetized samples. In this framework we investigate a 20 nm Fe film, hosting a self-organized hexagonal antidot lattice with *a* = 200 nm periodicity and *d* = 100 nm hole diameter. In careful combinations of longitudinal and polar MOKE measurements, which are shown in Figure 10 for the nn- and nnn-directions respectively, we find that a partial out-of-plane magnetisation arises from a purely in-plane applied field along the nn-direction of the antidot lattice. Furthermore, this perpendicular magnetisation component couples to the in-plane magnetisation as it develops and responds together with the measured in-plane component [57]. All fundamental models used to understand and describe the antidot based in-plane magnetisations of extended 20 nm thin iron films are related to thin film shape anisotropy, fixing the local magnetisation in the film plane. In the regime of nanoscaled antidot lattices, this is not simply valid anymore as the local “bridge” aspect ratio is reduced to only 5:1 in the investigated system. Hence, it is likely that the easy axis is not fixed to the iron thin film plane, allowing at least partially perpendicular components pointing out of the surface plane [57].

**Figure 11:**
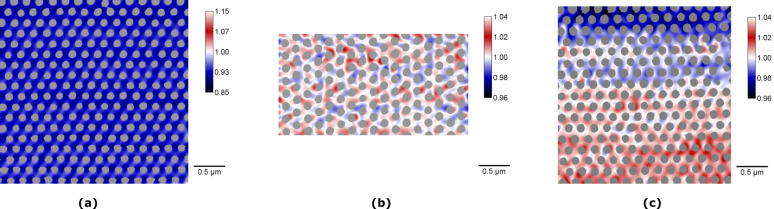
Fe *L*_3_ edge XMCD contrast of X-ray micrographs under normal incidence of a hexagonal antidot lattice with *a* = 200 nm antidot periodicity and *d* = 100 nm antidot diameter in a *t* = 20 nm thick Fe film (cf. Figure 2). An in-plane magnetic field of 240 mT is applied in horizontal direction, i.e., along the nn-direction for images (a) and (c) and along the nnn-direction for image (b). The positions of the holes are indicated as grey overlays. While panels (a) and (c) show the same structural orientation, panel (a) exhibits a homogeneously magnetized area and (c) features a domain wall. Reproduced with permission from [57] under CC-BY 3.0 license, copyright 2015 IOP Publishing Limited.

However, we only find non-vanishing out-of-plane MOKE contributions for magnetic fields applied along the nn-direction (Figure 10b), and not for fields applied along the nnn-direction (Figure 10d). We performed quantitative STXM microscopy, to further elucidate this behaviour. On a length scale down to the antidote lattice size, we found partial out-of-plane deflection for all orientations of the antidot lattice, as is shown in the centre of Figure 11. While it only occurs in larger micrometre-sized homogeneous domains for applied magnetic fields along the nn-direction, perpendicular regions are still present for fields applied along the nnn-direction, but chaotically fragment into up and down components. Thus, the resulting out-of-plane magnetisations are averaged out on the micrometre scale, leading to a vanishing polar Kerr effect as observed in MOKE measurements (Figure 10d) [57]. This difference is based on the different magnetisation reversal processes discussed above. The STXM results in Figure 11 provide the basis for a simple explanation of this phenomenon. While the magnetisation reversal along the nn-direction occurs via a large area domain wall movement, the magnetic switching along the nnn-direction occurs in fragmented rotation of individual small areas of the antidot lattice. These different types of magnetisation reversals are transferred to the out-of-plane component via Bloch-type domain walls, pulling the out-of-plane component into one direction, thus lifting the two-fold perpendicular degeneracy [57].

#### Magnetism of out-of-plane magnetized GdFe antidot films

The above findings of the rich switching properties in in-plane magnetized Fe films directly motivate investigations of out-of-plane magnetized films. We have chosen the well-known GdFe system [58] since it provides an amorphous, magnetically soft film with out-of-plane easy axis.

**Figure 12:**
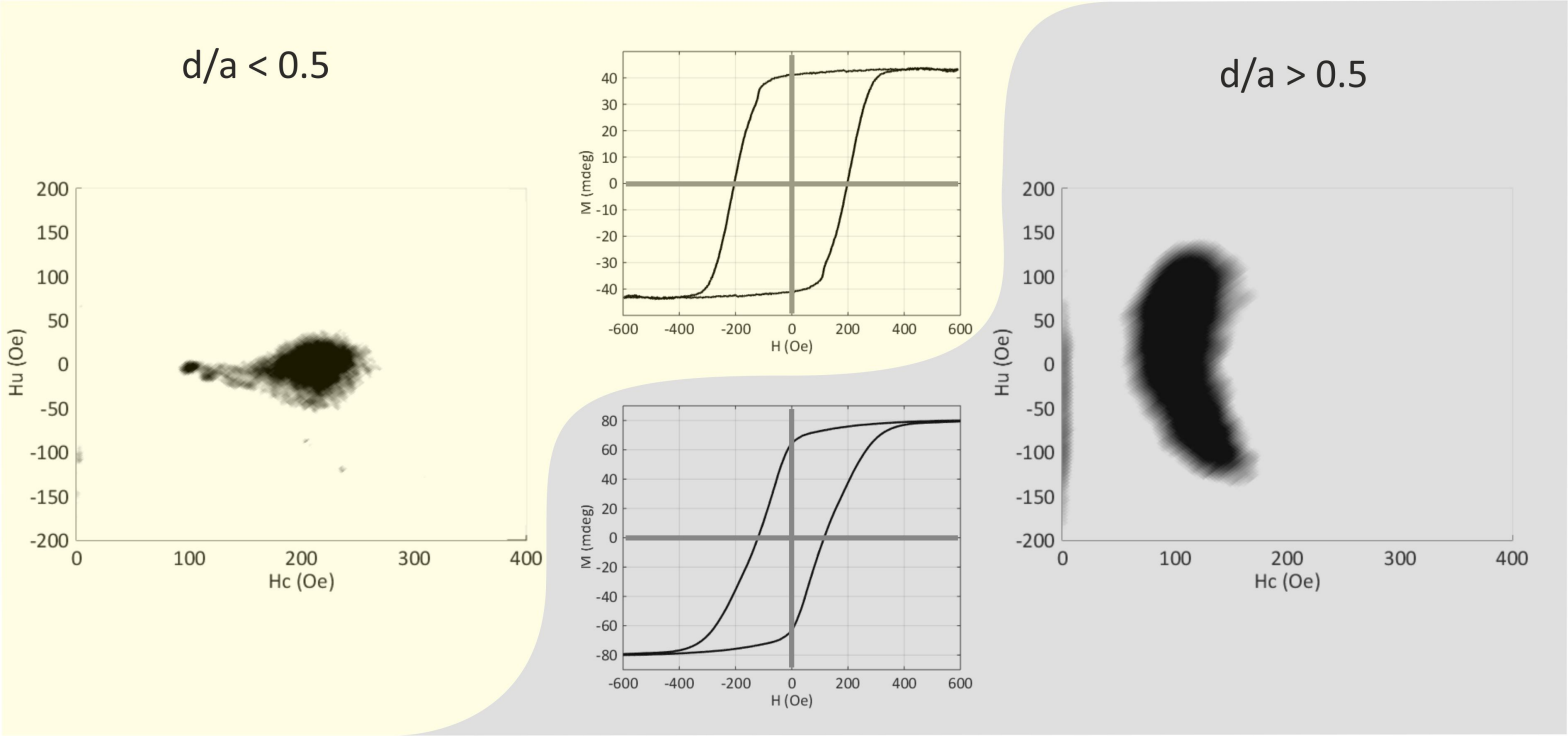
Major hysteresis loops and FORC diagrams of two hexagonal antidot lattices in out-of-plane magnetized GdFe films. Both feature the same hole spacing *a* = 200 nm, but the hole diameter *d* is 95 nm and 120 nm, respectively. Reproduced with permission from [59], copyright 2015 IEEE.

The impact of a nanostructured antidot lattice on in-plane magnetized films is naively understood by structural variations given by the perforations along the direction of the magnetisation. This is not as simple in case of perpendicularly magnetized thin films like thin GdFe multilayers. On the other hand, perpendicularly magnetized thin films provide strong out-of-plane demagnetizing fields. These are the origin of the often-observed labyrinth like domain patterns. Patterning reduced the effect of the demagnetizing field in a similar way as discussed above for in-plane fields, based on an effective local demagnetisation factor, which is strongly reduced as compared to the continuous thin film. Based on these arguments, we expect a reduced demagnetisation and therefore a more stable perpendicular magnetisation. In general accordance with that, we observe a significant impact of the antidot lattice on the magnetic properties of GdFe thin films. This becomes evident from the hysteresis loops shown in the centre of Figure 12 for GdFe antidot films with *d* = 95 nm and 120 nm at a period *a* = 200 nm.

Using the FORC method described above, we acquire a fingerprint of the magnetisation reversal processes of antidot lattices with large and small antidots in GdFe thin films. Based on FORC we furthermore deduced the microscopic influence of the antidot structuring, which strongly depends on the hole size. While the major hysteresis loops are somewhat similar, the FORC diagrams in Figure 12 exhibit fundamentally different features, showing that the underlying microscopic processes must be essentially different. The 95 nm antidot sample exhibits a rather broad distribution of the coercive field while the interaction field is almost zero. The 120 nm antidot sample, however, has a much wider distribution of interaction fields at overall lower coercive fields. With this information, the field region of interest can be set for STXM microscopy and the microscopic features can be identified (not shown). For the smaller antidots, the switching mode is dominated by domain wall pinning at antidots of slightly varying diameters, while for the larger antidots the bridges reverse individually although dipolar and exchange coupled via the common vertices. Thus, we can tune the interaction between bridges by changing the antidot diameter [59].

**Figure 13:**
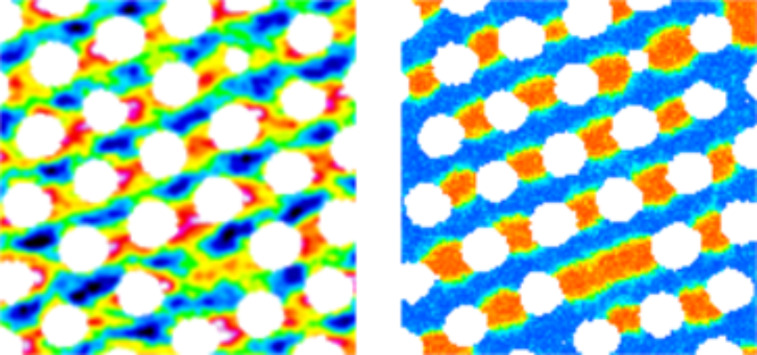
Left image: XMCD image of a 43 nm thin FeGd film with antidot diameter of 165 nm and centre-to-centre spacing of 200 nm. Right image: Results of a simulation of the same film geometry at *T* = 300 K. The saturation magnetisation was increased by 40% and the anisotropy was decreased by 30%, respectively.

XMCD images of thin GdFe films show, among other patterns, parallel stripe domains. One such domain configuration is shown in the left panel of Figure 13. We tried to reproduce this pattern in a simulation. Therefore, we directly adopt the antidot mask from the STXM to the simulations. The film dimensions and film thickness were matched to that of the real system as close as possible. The simulated film has an area of 1.28 × 1.28 µm^2^ and a thickness of 40 nm (compared to 43 nm in the XMCD image). The grain size is 10 × 10 × 10 nm^3^, resulting in a simulation mesh of 128 × 128 × 4 grains. The required input parameters were obtained and estimated from experiments. The temperature dependent magnetisation of the material was measured between 10 and 350 K in a 7 T magnetic field to ensure full saturation. A linear extrapolation of the magnetisation curve from 10 K down to 0 K delivered a saturation magnetisation of 4.26 × 10^5^ A/m leading to an estimated equilibrium magnetisation (normalized to the saturation magnetisation) at 300 K of 0.16. The uniaxial anisotropy of 0.05 emu Oe at 300 K was measured as the area under the difference of a perpendicular and an in-plane hysteresis loop of an unpatterned film with the dimensions 5 mm × 5 mm × 21.6 nm, which corresponds to an uniaxial anisotropy of 9.26 × 10^3^ J/m^3^. We obtain 3.62 × 10^5^ J/m^3^ for the uniaxial anisotropy at *T* = 0 K from a relation stating that the uniaxial anisotropy is proportional to the square of the equilibrium magnetisation [34–35],

[2]
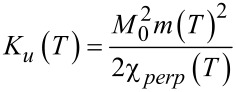


Where χ*_perp_*(*T*) is the perpendicular susceptibility, *M*_0_ is the saturation magnetization at *T* = 0 K and *m*(*T*) is the normalized magnetisation at a given temperature *T*. The exchange stiffness was estimated to 9.17 × 10^−11^ J/m from the domain wall width in the XMCD image, which is roughly 50 nm.

To investigate the stability of the experimentally observed stripe domains in the simulation, a stripe pattern was enforced as an initial condition. The system was then given time to relax. With the above material parameters the stripe domains completely disappeared after 1.4 ns. However, by increasing the saturation magnetisation by 40% and decreasing the uniaxial anisotropy by 30% further simulations yielded a stable stripe domain configuration (after 2 ns) as shown in Figure 13. The origin of these necessary parameter changes is not clear, but the following possibilities probably play a role. The first one is the experimental estimate of the exchange stiffness, which is proportional to the square of the domain wall width and, therefore, any error in this width is magnified. Another source of error may be the estimated uniaxial anisotropy at *T* = 0 K, since it is not clear to what extent the used formula applies to ferrimagnetic materials. Moreover, the reduction of GdFe to a ferromagnet with only one sublattice in the simulation could likewise contribute to the discrepancy.

## Conclusion

The magnetic behaviour of thin films with nanoscale antidot lattices in a range from 45 to 200 nm antidot diameters was investigated. Both, in-plane magnetized Fe, Co, and Py as well as out-of-plane magnetized GdFe antidot films were prepared by a modified nanosphere lithography allowing for non-close packed voids in a magnetic film. The combination of magnetometry, magneto-resistance measurements, and magnetic microscopy techniques as well as micromagnetic simulations delivered a detailed understanding of the occurring domain structures and switching modes.

Domain configurations, switching modes, and additional anisotropies can be controlled via the geometry of the antidot lattices. For antidots with a *d*/*a* ratio >0.75 we find domain configurations which obey the spin ice rules. For a *d*/*a* ratio <0.75 the switching mode and, with that, the coercivity of the sample depend on the orientation of the external field relative to the crystal axes of the antidot lattice. Furthermore, frustration effects can lead to significant out-of-plane magnetisation contributions during reversal. For FeGd films with out-of-plane magnetisation, the antidots can stabilize stripe domains, which can be imprinted via an initial saturation with the stripes along that nearest-neighbour direction of the antidot lattice, which is closest to the saturating field. Over all, antidot lattices offer a variety of possibilities to design the magnetic properties of thin films for e.g. the application as spin-wave filters or artificial spin-ice structures.

## Experimental

Antidot samples were prepared by PS-based nanosphere lithography. Surfactant-free latex sphere dispersions with nominal sphere diameters of 100 nm, 200 nm, and 500 nm were purchased from Life Technologies Ltd. [60] and diluted with millipore water to 1% w/v. Depending on the needs of the various magnetic characterisation techniques, different substrates were used: (a) a 300 nm thick, thermally grown SiO_2_ layer on Si substrate for magnetotransport measurements, (b) a 0.5 × 0.5 mm^2^ Si_3_N_4_ membrane with a thickness of 500 nm for STXM purchased from Silson Ltd. [61]. For all substrates, we increased the hydrophilicity for improved self-assembly of PS spheres by exposing the substrates to an oxygen plasma (DC bias voltage at −80 V for 5 minutes) [62]. Then, PS spheres were deposited by dip coating on the substrates using a computer-controlled motor driving the linear motion of the substrates at an angle of 60° with respect to dispersion surface. The extraction velocity (about 10 µm/s) is the most critical parameter for the process and optimized for each PS sphere dispersion and concentration at given temperature and humidity in the laboratory. For the plasma etching, an Oxford Plasma Technology Type 80Plus combined RIE (Reactive Ion Etching) and ICP (inductively coupled plasma) source was used [12]. The size reduction of the PS spheres was done in an oxygen plasma with a 25 W RIE component and 100 W ICP component at a DC bias voltage of −80 V. For PS spheres with an initial diameter of 200 nm, the achieved diameter as function of etching time has been determined in detail in advance [12,14]. Due to the plasma’s anisotropic component, PS particles lost their spherical shape and became oblate. This, however, does not affect their function as templates for antidots.

Fe, Co and Py (Ni_81_Fe_19_) films were deposited and covered by 2 nm Pt to prevent oxidation by pulsed laser deposition (PLD) under ultrahigh vacuum conditions. Details of the deposition system can be found elsewhere [63]. All of these materials have low coercive fields in common with strengths below 20 Oe at 300 K and an in-plane easy axis of magnetisation. GdFe multilayer films [0.36 nm / 0.36 nm] were deposited with a 2 nm Al capping layer under UHV conditions by ion beam sputtering [58,64]. GdFe multilayer films feature low coercive fields and an out-of-plane easy axis of magnetisation. The total film thickness was adjusted from 29 nm (*d*/*a* < 0.5) to 45 nm (*d*/*a* > 0.5) to allow reliable PS sphere template lift off [64].

PS spheres including the magnetic caps were removed by manually rubbing the nanostructured surface under a small force on an acetone soaked stash of lens cleaning paper for one minute. The result of the above processing is displayed in Figure 2. An alternative method to remove caps and PS spheres is the application of ultrasound in acetone, applicable, however, only to massive substrates.
